# Association of novel and conventional obesity indices with colorectal cancer risk in older Chinese: a 14-year follow-up of the Guangzhou Biobank Cohort Study

**DOI:** 10.1186/s12885-023-10762-0

**Published:** 2023-03-29

**Authors:** Shu Yi Wang, Wei Sen Zhang, Chao Qiang Jiang, Ya Li Jin, Tong Zhu, Feng Zhu, Lin Xu

**Affiliations:** 1 School of Public Health, Sun Yat-sen University, No. 74 Zhongshan 2nd Road, Guangzhou, Guangdong Province China; 2 Guangzhou Twelfth People’s Hospital, Guangzhou, China; 3 School of Public Health, the University of Hong Kong, Hong Kong, China

**Keywords:** Obesity, Visceral adiposity index, A body shape index, Colorectal cancer

## Abstract

**Background:**

Visceral adiposity index (VAI) and a body shape index (ABSI) were newly developed indices for visceral fat mass. Whether they are superior to conventional obesity indices in predicting colorectal cancer (CRC) remains unclear. We examined the associations of VAI and ABSI with CRC risk, and investigated their performance in discriminating CRC risk compared with conventional obesity indices in the Guangzhou Biobank Cohort Study.

**Methods:**

A total of 28,359 participants aged 50 + years without cancer history at baseline (2003-8) were included. CRC were identified from the Guangzhou Cancer Registry. Cox proportional hazards regression was used to assess the association of obesity indices with the CRC risk. Discriminative abilities of obesity indices were assessed using Harrell’s C-statistic.

**Results:**

During an average follow-up of 13.9 (standard deviation = 3.6) years, 630 incident CRC cases were recorded. After adjusting for potential confounders, the hazard ratio (95% confidence interval) of incident CRC for per standard deviation increment in VAI, ABSI, body mass index (BMI), waist circumference (WC), waist-to-hip ratio (WHR) and waist-to-height ratio (WHtR) was 1.04 (0.96, 1.12), 1.13 (1.04, 1.22), 1.08 (1.00, 1.17), 1.15 (1.06, 1.24), 1.16 (1.08, 1.25)and 1.13 (1.04, 1.22), respectively. Similar results for colon cancer were found. However, the associations of obesity indices with risk of rectal cancer were non-significant. All obesity indices showed similar discriminative abilities (C-statistics from 0.640 to 0.645), with WHR showing the highest whilst VAI and BMI the lowest.

**Conclusions:**

ABSI, but not VAI, was positively associated with a higher risk of CRC. However, ABSI was not superior to the conventional abdominal obesity indices in predicting CRC.

**Supplementary Information:**

The online version contains supplementary material available at 10.1186/s12885-023-10762-0.

## Introduction

Colorectal cancer (CRC) is the third most common cancer and the second leading cause of cancer death worldwide, with an estimated 1.9 million new cases and 935,000 death in 2020[1]. The incidence of CRC has been increasing over the last three decades, especially in low-and middle-income [2], which can be partly attributable to changes in lifestyles and some modifiable risk factors, further highlighting the importance of primary prevention.

Obesity is an established risk factor of CRC [3, 4]. Body mass index (BMI) and waist circumference (WC) are two most widely used conventional measures for obesity. However, BMI cannot indicate fat [5] while WC cannot distinguish between subcutaneous and visceral fat [6]. As it is suggested that visceral adipose tissue was more strongly associated with colorectal adenomas than subcutaneous adipose tissue [7], increasing research efforts have been made on identifying alternative obesity indices that have stronger predictive value for visceral fat mass.

Some alternative indices of visceral obesity have been introduced [8–14]. Of them, visceral adiposity index (VAI) [8] and a body shape index (ABSI) [9] were most commonly used in epidemiologic studies to examine the associations with all-cause mortality, cardiovascular disease or diabetes [15–17]. As both VAI and ABSI account for WC, height and weight simultaneously, they were suggested to have better discrimination of visceral adipose mass and distribution [8, 9]. Moreover, studies based on magnetic resonance imaging indicated that VAI and ABSI had significant correlation with visceral adipose [8, 18]. A recent study in Japan showed a positive association between VAI and the risk of CRC [19]. Furthermore, higher ABSI was also associated with a higher risk of CRC [20–22]. High ABSI corresponds to a greater level of visceral adipose tissue at a given height and weight, which may secrete more proinflammatory adipokines and is more heavily infiltrated with immune cells [23, 24], contributing to colon cancer development [25]. Meanwhile, the immune cells activated by adipose tissue can also promote insulin resistance and subsequent hyperinsulinemia [26], which may increase CRC risk by inducing tumor cell proliferation and decreasing apoptosis [27, 28]. However, no consensus was made in the predicting ability of VAI or ABSI with comparison to conventional obesity indices. A pooled collaborative analysis of 11 Australian cohorts reported similar predictive ability of CRC between ABSI and other obesity indices in both men and women [29] whereas another recent study from Sweden found that although ABSI had a weaker association with colon cancer than WC in both sexes, ABSI but not WC predicted the risk of rectal cancer significantly in men [30]. The discrepancies between the studies highlight the need for further research to better understand the discriminatory ability for each index, specifically among older people with a higher risk of CRC.

Therefore, we examined the associations of VAI and ABSI with CRC risk in middle-aged to older Chinese using data from the Guangzhou Biobank Cohort Study, and compared their performance in predicting CRC risk with conventional obesity indices, including body mass index (BMI), waist circumference (WC), waist-to-hip ratio (WHR) and waist-to-height ratio (WHtR).

## Methods

### Study participants

Guangzhou Biobank Cohort Study (GBCS) is a 3-way collaboration among Guangzhou Twelfth People’s Hospital and the Universities of Hong Kong and Birmingham. Details of GBCS have been reported elsewhere [31]. In brief, all participants were recruited during 2003 to 2008 from the Guangzhou Health and Happiness Association for the Respectable Elders (GHHARE), which was a community social and welfare organization unofficially aligned with the municipal government. GHHARE included about 7% of Guangzhou residents in this age group, with branches in all districts of Guangzhou. Membership was open to Guangzhou residents aged 50 years or above for a minimal nominal fee of ¥4 (about 50 US cents) per month. The baseline examination included a face-to-face computer-assisted interview by trained nurses on demographic characteristics, lifestyle factors, family and personal medical history and assessment of anthropometric parameters, blood pressure, fasting plasma glucose and lipids. Reliability and validity of the questionnaire were tested 6 months into recruitment by recalling 200 randomly selected participants for re-interview [31]. Ethics approval was granted by the Guangzhou Medical Ethics Committee of the Chinese Medical Association and all participants provided written, informed consent before participation.

### Obesity indices

Anthropometric measurements were performed by trained nurses in the morning before breakfast following standard protocols, including weight, standing height, WC and hip circumference (HC) with light indoor clothing and without shoes. BMI was calculated as weight in kilogram divided by height in meter squared (kg/m^2^). WC was measured horizontally around the smallest circumference between the ribs and iliac crest, or at the navel, if no natural waistline was present. HC was measured around the maximal girth of the hips. WHR was calculated by dividing WC (cm) by HC (cm), and WHtR was calculated by dividing WC (cm) by height (cm). ABSI was derived using the formula (a), with WC and height expressed in meter [9], as follows:

ABSI = WC/(BMI^2/3^ × height^1/2^) (a).

VAI in men and women was calculated using the following formula (c) and (d), respectively, where WC were expressed in cm, and triglycerides (TG) and high-density lipoprotein cholesterol (HDL-c) in mmol/L [8]:

VAI = (WC/(39.68 + 1.88 × BMI)) × (TG/1.03) × (1.31/HDL-c) (c).

VAI = (WC/(36.58 + 1.89 × BMI)) × (TG/0.81) × (1.52/HDL-c) (d).

The Chinese-specific cut-offs for general obesity, as recommended by the World Health Organization (WHO), were used, with underweight defined as BMI lower than 18.5 kg/m^2^, normal weight 18.5–24.9 kg/m^2^, overweight 25.0–27.4 kg/m^2^ and general obesity ≥ 27.5 kg/m^2 ^[32]. Higher WC was defined according to Asian-specific cut-offs suggested by WHO with ≥ 90 cm for men and ≥ 80 cm for women [33]. Higher WHR and WHtR were defined by established cut-offs, i.e., men ≥ 0.9 and women ≥ 0.8 for WHR [34] and ≥ 0.5 for WHtR [35]. As no established cut-offs for VAI and ABSI, they were categorized into tertiles.

### Outcomes

The study outcome was incident CRC (Tenth Revision of the International Classification of Diseases (ICD-10) C18-C20). Information on first diagnosis of CRC up to April 2021 was obtained from the Guangzhou Cancer Registry (GCR) of the Guangzhou Center for Disease Control and Prevention (GZCDC).

### **Potential** confounders

As age [36], sex [37], lifestyle factors (physical activity [38], smoking [39, 40], alcohol drinking [41, 42], consumption of vegetable, fruits and red meat [43]) were associated with both obesity and CRC, these factors were considered as potential confounders and adjusted in the Cox regression models. Furthermore, education and household annual income were also included to partly account for confounding due to socioeconomic position [44]. Physical activity was categorized into inactive, moderate and active based on the short version of the International Physical Activity Questionnaire (IPAQ), which has been validated previously [45]. Consumption of vegetable, fruits and red meat intake were assessed using a validated food frequency questionnaire (FFQ), which covered the average frequency over the last seven days and amount of consumption of each item [46]. Vegetable, fruits and red meat intake was calculated by multiplying the frequency by the amount of intake each time.

### **Potential** mediators

As chronic inflammation may involve in the pathway between obesity and development of CRC [47], we also assessed the potential mediating effects due to white blood cell count (WBC), a reliable biomarker for inflammation [48, 49]. WBC were assayed in an automated hematology analyzer (KX-21, SYSMEX, Japan) [31].

### Statistical analysis

Differences of distribution in demographic and other characteristics by incident CRC were examined using t-test or Wilcoxon test for continuous variables and chi-square test for categorical variables. The correlations among ABSI, VAI and conventional obesity indices were evaluated using Pearson correlation coefficients (Pearson’s *r*) by sex. To enable comparisons between different obesity indices with different units, all indices were standardized using z-scores, calculated by the following equation (e). Sex-specific z-scores was used for WC and WHR.

z-score = (actual value-mean)/standard deviation (SD) (e).

Cox proportional hazard regression was used to estimate crude and adjusted hazard ratio (HR) and 95% confidence intervals (CI) for CRC risk from different obesity indices. The proportional hazard assumption was tested by the Schoenfeld residual method. No evidence for the violation of the assumption was found. Furthermore, the potential nonlinear relationships between obesity indices and risk of CRC were estimated by using restricted cubic splines with three knots (10th, 50th, 90th percentiles) based on Cox regression. Potential interactions between obesity indices and sex or selected lifestyle factors including smoking, alcohol drinking and physical activity were tested by comparing model fit with and without the interaction terms using likelihood ratio test. To investigate the potential mediation of inflammation in the association between obesity and CRC, WBC were further adjusted in Cox regression model. The discriminative abilities of the obesity indices for CRC were assessed using Harrell’s C-statistic. Sensitivity analyses were conducted by excluding participants with less than 5 years of follow-up and restricting analyses to never-smokers to limit possible reverse causality and residual confounding. All analyses were performed using R software (version 4.1.3) and all tests were two-sided with a significant level of *P* < 0.05.

## Results

At baseline, a total of 30,430 participants were recruited. After excluding those with self-reported cancer at baseline (n = 585), missing information on anthropometric measures (n = 301) or other covariates (n = 733) and loss to follow-up for vital status (n = 452), 28,359 (72.3% women) were included in the current analyses. The mean age was 62.0 (SD = 7.1) years. During an average follow-up of 13.9 (SD = 3.6) years, 630 (61.3% women) incident CRC were recorded.

Table 1 shows that participants diagnosed with CRC were older, had higher proportion of men and had higher levels of WC, WHR, WHtR and ABSI (all *P* < 0.05). No significant association was found between other characteristics (education, household annual income, alcohol drinking, smoking, physical activity, vegetable intake, fruit intake, red meat intake, WBC, TG, HDL-c, BMI and VAI) and incident CRC (*P* > 0.05). ABSI was weakly correlated with WHtR and VAI (Pearson’s *r* from 0.06 to 0.16), but moderately correlated with BMI, WHR and WC (Pearson’s *r* from 0.54 to 0.71) (Supplementary Table 1). VAI had weak correlations with conventional obesity indices (Pearson’s *r* from 0.17 to 0.27) (Supplementary Table 1). Table 2 shows that, after adjustment for age, sex, smoking, alcohol drinking, household annual income, education, physical activity, intake of vegetable, fruits and red meat, per SD increment in BMI, WC, WHR, WHtR and ABSI was significantly associated with higher CRC risk, with HR (95% CI) being 1.08 (1.00, 1.17), 1.15 (1.06, 1.24), 1.16 (1.08, 1.25), 1.13 (1.04, 1.22) and 1.13 (1.04, 1.22), respectively. No significant association was found between per SD increment in VAI and CRC risk. The results were similar for colon cancer, with HR (95% CI) for per SD increment in BMI, WC, WHR, WHtR, VAI and ABSI being 1.11 (1.01, 1.21), 1.18 (1.07, 1.29), 1.21 (1.10, 1.33), 1.16 (1.06, 1.28), 1.06 (0.97, 1.15) and 1.15 (1.04, 1.26), respectively (Table 3). However, non-significant associations of obesity indices with the development of rectal cancer were found, with adjusted HR for per SD increment in BMI, WC, WHR, WHtR, VAI and ABSI being 1.04 (0.90, 1.19), 1.09 (0.95, 1.25), 1.07 (0.93, 1.23), 1.06 (0.92, 1.23), 0.98 (0.85, 1.15) and 1.08 (0.94, 1.25), respectively (Table 3). Similar results were found for risk of CRC and colon cancer when obesity indices were analyzed as categorical variables (Tables 2 and 3). Sensitivity analyses by excluding participants with smoking history (i.e., former and current smokers) and less than 5 years of follow-up yielded similar results (Supplementary Tables 2 to 5).

**Table 1:** Baseline characteristics of participants by colorectal cancer (CRC)

	Total	CRC		*P* value
		No	Yes	
Number	28,359	27,729	630	-
Age (years), mean (SD)	62.0 (7.11)	62.0 (7.10)	64.6 (6.73)	< 0.001
Sex, N (%)				< 0.001
Men	7,848 (27.7)	7,604 (27.4)	244 (38.7)	
Women	20,511 (72.3)	20,125 (72.6)	386 (61.3)	
Education, N (%)				0.14
Primary school or below	12,219 (43.1)	11,943 (43.1)	276 (43.8)	
Middle school	13,641 (48.1)	13,355 (48.2)	286 (45.4)	
College or above	2,499 (8.8)	2,431 (8.8)	68 (10.8)	
Household annual income (yuan), N (%)				0.25
< 10,000	1,615 (5.7)	1,576 (5.7)	39 (6.2)	
≥ 10,000~<30,000	9,148 (32.3)	8,945 (32.3)	203 (32.2)	
≥ 30,000~<50,000	6,005 (21.2)	5,891 (21.2)	114 (18.1)	
≥ 50,000	4,796 (16.9)	4,691 (16.9)	105 (16.7)	
Not known	6,795 (24.0)	6,626 (23.9)	169 (26.8)	
Alcohol drinking in woman, N (%)				
Never	16,265 (79.3)	15,949 (79.2)	316 (81.9)	0.22
Former	518 (2.5)	513 (2.5)	5 (1.3)	
Current	3,728 (18.2)	3,663 (18.2)	65 (16.8)	
Alcohol drinking in man, N (%)				0.82
Never	4,251 (54.2)	4,114 (54.1)	137 (56.1)	
Former	478 (6.1)	464 (6.1)	14 (5.7)	
Current	3,119 (39.7)	3,026 (39.8)	93 (38.1)	
Smoking in woman, N (%)				
Never	19,788 (96.5)	19,421 (96.5)	367 (95.1)	0.32
Former	341 (1.7)	332 (1.6)	9 (2.3)	
Current	382 (1.9)	372 (1.8)	10 (2.6)	
Smoking in man, N (%)				
Never	3,144 (40.1)	3,046 (40.1)	98 (40.2)	0.88
Former	2,222 (28.3)	2,150 (28.3)	72 (29.5)	
Current	2,482 (31.6)	2,408 (31.7)	74 (30.3)	
Physical activity, N (%)				0.23
Inactive	2,270 (8.0)	2,227 (8.0)	43 (6.8)	
Moderate	11,526 (40.6)	11,251 (40.6)	275 (43.7)	
Active	14,563 (51.4)	14,251 (51.4)	312 (49.5)	
Vegetable intake (g/day), median (IQR)	228.6 (143.6, 347.7)	228.6 (143.6, 347.1)	223.6 (146.3, 349.0)	0.94
Fruit intake (g/day), median (IQR)	122.9 (58.9, 200.0)	122.9 (58.9, 201.4)	125.0 (59.2, 187.5)	0.82
Red meat intake (g/day), median (IQR)	42.9 (25.0, 60.7)	42.9 (25.0, 60.7)	39.3 (25.0, 62.5)	0.70
White blood cell (*10^9^/L), mean (SD)	6.35 (1.59)	6.35 (1.59)	6.46 (1.48)	0.13
Triglycerides (mmol/L), mean (SD)	1.68 (1.26)	1.68 (1.26)	1.69 (1.13)	0.89
High-density lipoprotein-cholesterol (mmol/L), mean (SD)	1.66 (0.40)	1.66 (0.40)	1.64 (0.40)	0.24
Adiposity indices				
Body mass index (kg/m^2^), mean (SD)	23.8 (3.31)	23.8 (3.31)	24.0 (3.38)	0.16
Waist circumference (cm), mean (SD)	78.9 (8.99)	78.8 (8.98)	80.8 (9.18)	< 0.001
Waist-to-hip ratio, mean (SD)	0.87 (0.07)	0.87 (0.07)	0.89 (0.07)	< 0.001
Waist-to-height ratio, mean (SD)	0.50 (0.06)	0.50 (0.06)	0.51 (0.06)	< 0.001
Visceral adiposity index, mean (SD)	1.85 (1.85)	1.85 (1.86)	1.83 (1.63)	0.83
A body shape index, mean (SD)	0.0764 (0.00)	0.0764 (0.00)	0.0776 (0.00)	< 0.001

**Table 2:** Association of adiposity indices with the risk of total colorectal cancer on 28,359 participants

	Incidence rate (per 1000 person-years)	Crude HR (95% CI)	Adjusted HR (95% CI)^†^
Body mass index (kg/m^2^)			
<18.5	1.68	1.08 (0.74, 1.59)	0.98 (0.66, 1.44)
≥18.5 ~ < 25.0	1.56	1.00	1.00
≥25.0 ~ < 27.5	1.61	1.03 (0.85, 1.26)	1.05 (0.86, 1.28)
≥27.5	1.78	1.14 (0.91, 1.44)	1.20 (0.95, 1.51)
z-score (1 SD = 3.31)		1.05 (0.97, 1.14)	1.08 (1.00, 1.17)^*^
Waist circumference (cm)			
<80 for W;<90 for M	1.54	1.00	1.00
≥80 for W; ≥90 for M	1.73	1.12 (0.96, 1.32)	1.18 (1.00, 1.40)
z score (1 SD = 8.70 for W;9.08 for M)		1.20 (1.11, 1.29)^***^	1.15 (1.06, 1.24)^***^
Waist-to-hip ratio			
<0.8 for W;<0.9 for M	1.39	1.00	1.00
≥0.8 for W; ≥0.9 for M	1.67	1.20 (0.99, 1.45)	1.40 (1.15, 1.71)^**^
z score (1 SD = 0.07 for W;0.06 for M)		1.23 (1.14, 1.32)^***^	1.16 (1.08, 1.25)^***^
Waist-to-height ratio			
< 0.5	1.40	1.00	1.00
≥ 0.5	1.77	1.27 (1.08, 1.49)^**^	1.16 (0.99, 1.37)
z-score (1 SD = 0.06)		1.18 (1.09, 1.27)^***^	1.13 (1.04, 1.22)^**^
Visceral adiposity index			
Tertile 1 (< 1.05)	1.57	1.00	1.00
Tertile 2 (≥ 1.05 ~ < 1.83)	1.51	0.96 (0.79, 1.17)	1.02 (0.84, 1.25)
Tertile 3 (≥ 1.83)	1.73	1.10 (0.91,1.33)	1.25 (1.03, 1.52)^*^
z-score (1 SD = 1.85)		0.99 (0.92, 1.08)	1.04 (0.96, 1.12)
A body shape index			
Tertile 1 (< 0.0742)	1.12	1.00	1.00
Tertile 2 (≥ 0.0742 ~ < 0.0784)	1.55	1.39 (1.13, 1.72)^**^	1.13 (0.91, 1.41)
Tertile 3 (≥ 0.0784)	2.16	1.95 (1.60, 2.38)^***^	1.34 (1.09, 1.67)^**^
z-score (1 SD = 0.0050)		1.25 (1.18, 1.33)^***^	1.13 (1.04, 1.22)^**^

^†^Adjusting for age, sex, smoking, alcohol drinking, household annual income, education, physical activity, intake of vegetable, fruits and red meat

^*^: *P* < 0.05; ^**^: *P* < 0.01; ^***^: *P* < 0.001

**Table 3:** Association of adiposity indices with the risk of colon and rectal cancer on 28,359 participants

Cancer type	Colon cancer			Rectal cancer		
	Incidence rate (per 1000 person-years)	Crude HR (95% CI)	Adjusted HR (95% CI)^†^	Incidence rate (per 1000 person-years)	Crude HR (95% CI)	Adjusted HR (95% CI)^†^
Body mass index (kg/m^2^)						
<18.5	1.14	1.07 (0.67, 1.70)	0.96 (0.60, 1.53)	0.54	1.12 (0.57, 2.20)	1.01 (0.51, 1.99)
≥18.5 ~ < 25.0	1.07	1.00	1.00	0.49	1.00	1.00
≥25.0 ~ < 27.5	1.00	0.94 (0.73, 1.20)	0.95 (0.74, 1.21)	0.61	1.25 (0.90, 1.74)	1.29 (0.93, 1.79)
≥27.5	1.26	1.18 (0.89, 1.55)	1.23 (0.93, 1.62)	0.52	1.07 (0.70, 1.64)	1.13 (0.73, 1.73)
z-score (1 SD = 3.31)		1.08 (0.98, 1.18)	1.11 (1.01, 1.21)^*^		1.01 (0.87, 1.15)	1.04 (0.90, 1.19)
Waist circumference (cm)						
<80 for W;<90 for M	1.02	1.00	1.00	0.52	1.00	1.00
≥80 for W; ≥90 for M	1.21	1.18 (0.97, 1.44)	1.23 (1.00, 1.50)	0.52	1.01 (0.76, 1.35)	1.09 (0.81, 1.48)
z score (1 SD = 8.70 for W;9.08 for M)		1.23 (1.13, 1.36)^***^	1.18 (1.07, 1.29)^***^		1.12 (0.97, 1.28)	1.09 (0.95, 1.25)
Waist-to-hip ratio						
<0.8 for W;<0.9 for M	0.87	1.00	1.00	0.52	1.00	1.00
≥0.8 for W; ≥0.9 for M	1.15	1.32 (1.04, 1.68)^*^	1.53 (1.19, 1.97)^***^	0.52	0.99 (0.72, 1.36)	1.18 (0.84, 1.65)
z score (1 SD = 0.07 for W;0.06 for M)		1.28 (1.18, 1.39)^***^	1.21 (1.10, 1.33)^***^		1.11 (0.97, 1.27)	1.07 (0.93, 1.23)
Waist-to-height ratio						
< 0.5	0.93	1.00	1.00	0.47	1.00	1.00
≥ 0.5	1.21	1.30 (1.07, 1.58)^**^	1.16 (0.95, 1.42)	0.56	1.20 (0.91, 1.59)	1.16 (0.87, 1.54)
z-score (1 SD = 0.06)		1.23 (1.12, 1.35)^***^	1.16 (1.06, 1.28)^**^		1.08 (0.94, 1.24)	1.06 (0.92, 1.23)
Visceral adiposity index						
Tertile 1 (< 1.05)	1.02	1.00	1.00	0.55	1.00	1.00
Tertile 2 (≥ 1.05 ~ < 1.83)	1.06	1.03 (0.81, 1.31)	1.10 (0.86, 1.40)	0.46	0.82 (0.59, 1.16)	0.89 (0.63, 1.26)
Tertile 3 (≥ 1.83)	1.18	1.16 (0.92, 1.46)	1.32 (1.04, 1.67)^*^	0.55	1.00 (0.72, 1.38)	1.13 (0.81, 1.58)
z-score (1 SD = 1.85)		1.01 (0.93, 1.11)	1.06 (0.97, 1.15)		0.94 (0.80, 1.11)	0.98 (0.85, 1.15)
A body shape index						
Tertile 1 (< 0.0742)	0.74	1.00	1.00	0.38	1.00	1.00
Tertile 2 (≥ 0.0742 ~ < 0.0784)	1.03	1.40 (1.08, 1.82)^*^	1.11 (0.85, 1.45)	0.52	1.37 (0.95, 1.98)	1.17 (0.81, 1.70)
Tertile 3 (≥ 0.0784)	1.50	2.05 (1.60, 2.61)^***^	1.36 (1.05, 1.76)^*^	0.66	1.76 (1.24, 2.50)^**^	1.32 (0.91, 1.92)
z-score (1 SD = 0.0050)		1.28 (1.20, 1.36)^***^	1.15 (1.04, 1.26)^**^		1.20 (1.07, 1.35)^**^	1.08 (0.94, 1.25)

^†^Adjusting for age, sex, smoking, alcohol drinking, household annual income, education, physical activity, intake of vegetable, fruits and red meat

^*^: *P* < 0.05; ^**^: *P* < 0.01; ^***^: *P* < 0.001

**Fig. 1:**
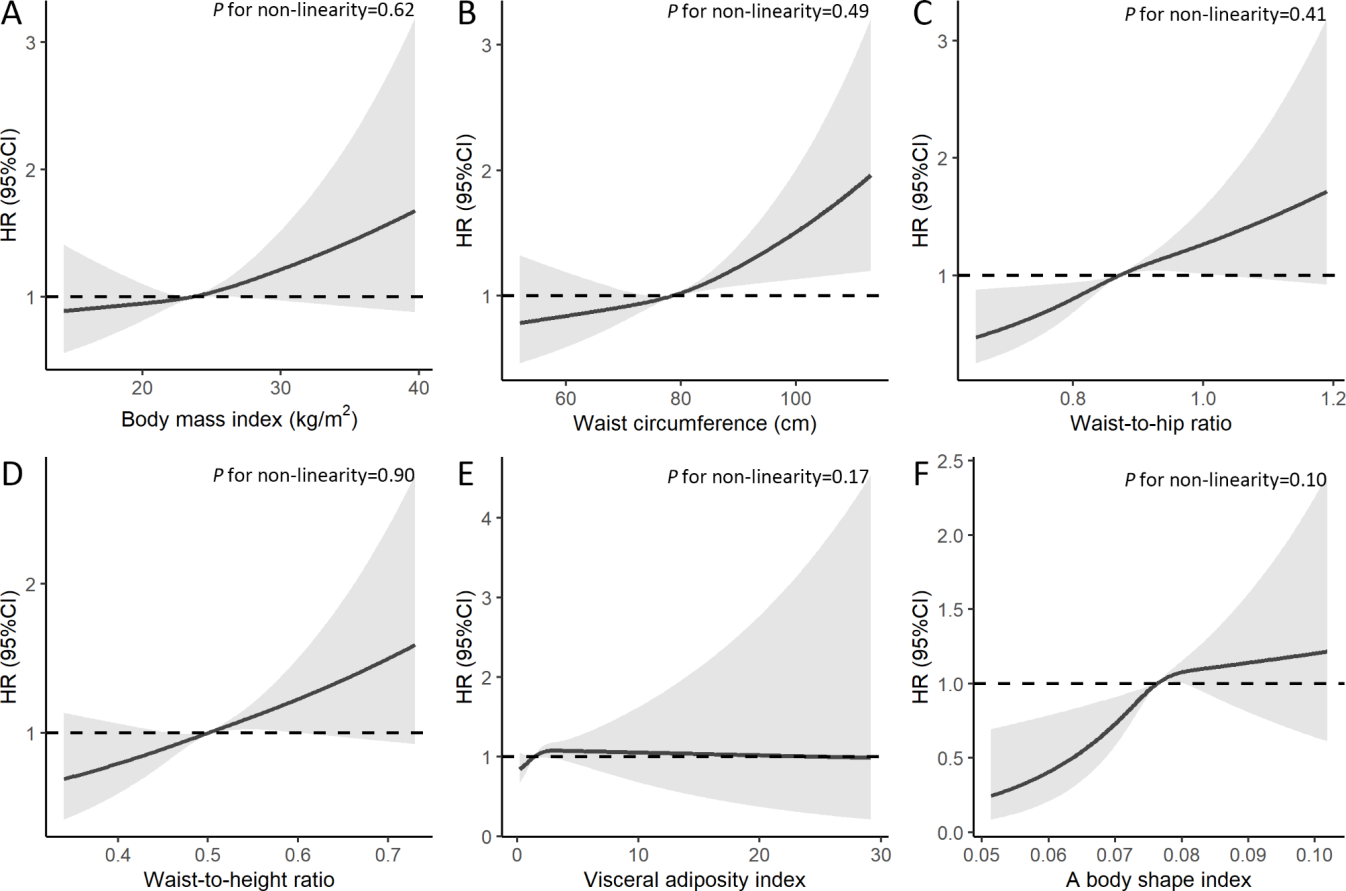
**Association of baseline adiposity indices (2003-8) with risk of colorectal cancer on 28,359 participants** Note: Potential nonlinear relationships were examined using restricted cubic splines (three knots on 10th, 50th and 90th ), with hazard ratio (HRs) from Cox proportional hazard models. The HRs was adjusted for age, sex, smoking, alcohol drinking, household annual income, education, physical activity, intake of vegetable, fruits and red meat

Table 4 shows the Harrell’s C-statistic of predicting CRC for all obesity indices ranging from 0.640 to 0.645. No significant difference in the C-statistic for CRC, colon and rectal cancers, respectively, across different obesity indices was found (*P* > 0.05). Results from the restricted cubic spline regression did not support the non-linearity assumptions between obesity indices and risk of CRC (all *P* for non-linearity > 0.05, Fig. 1). No significant interaction between obesity and potential moderators (sex, smoking, alcohol drinking and physical activity) was found (*P* for interaction from 0.15 to 0.89). The combination of general obesity (BMI) and each of the other obesity indices did not improve the predictive performance compared with each of indices alone (Supplementary Tables 6 to 8). The associations between obesity indices and CRC attenuated slightly after adjusting for WBC in the Cox regression models (Supplementary Table 9).

**Table 4:** Comparison of the discriminative ability of obesity indices for the prediction of colorectal cancer

	Harrell’s C statistic (95% CI) for obesity indices		
	Colorectal cancer	Colon cancer	Rectal cancer
Model 1	0.640 (0.618, 0.662)	0.658 (0.633, 0.683)	0.618 (0.577, 0.659)
Model 1 + Body mass index	0.640 (0.618, 0.662)	0.660 (0.635, 0.685)	0.618 (0.577, 0.659)
Model 1 + Waist circumference	0.644 (0.622, 0.666)	0.663 (0.638, 0.688)	0.622 (0.581, 0.663)
Model 1 + Waist-to-hip ratio	0.645 (0.623, 0.666)	0.667 (0.642, 0.692)	0.620 (0.579, 0.661)
Model 1 + Waist-to-height ratio	0.643 (0.621, 0.665)	0.662 (0.637, 0.687)	0.619 (0.578, 0.660)
Model 1 + Visceral adiposity index	0.640 (0.618, 0.662)	0.660 (0.635, 0.685)	0.618 (0.577, 0.659)
Model 1 + A body shape index	0.644 (0.622, 0.666)	0.663 (0.638, 0.688)	0.621 (0.580, 0.662)

Model 1: age, sex, smoking, alcohol drinking, household annual income, education, physical activity, intake of vegetable, fruits and red meat

## Discussion

In this large population-based prospective cohort study, we found that ABSI, but not VAI were associated with increased risk of total CRC and colon cancer. The predictive ability of VAI or ABSI was not superior to the conventional visceral adiposity indices (i.e., WC, WHR and WHtR). To our knowledge, our study is the first to systematically compare the ability of VAI/ABSI and conventional obesity indices in predicting CRC risk. As ABSI and the other indices related to central obesity appeared to provide similar prediction for CRC risk, simple measures such as WC or WHR are recommended.

Obesity is one of the important modifiable risk factors for CRC [50]. The gold standard measurement of abdominal obesity was by computerized tomography (CT) or magnetic resonance imaging (MRI). However, they were costly, time consuming and not practical for large epidemiologic studies [51]. Therefore, some simple clinical anthropometric indices were proposed as alternative ways for measuring visceral fat mass, such as VAI and ABSI. We found three studies reported significant positive associations of VAI and ABSI with CRC risk in Japan [19] and United Kingdom [20, 21], respectively. The association of VAI with CRC risk in our study appeared to be lower to that from the Japanese cohort (NAGALA study), i.e., participants with the highest tertile of VAI scores had 25% higher risk of CRC in our study versus 78% in the NAGALA study [19]. The discrepancy could be due to different reference groups used and various follow-up duration. Notably, as the NAGALA study included 27,921 middle-aged adults (mean age = 45.7 years) with relatively short follow-up duration (4.4 years), the number of incident CRC events was relatively small [19]. Our study found that per SD increment in ABSI was associated with 13% greater risk of CRC, which was generally consistent with previous studies. Two studies using data from the UK Biobank with followed-up of 7 years reported that per SD higher ABSI was associated with a higher risk of CRC by 16% in men, 7% in women [20] and 9% in total [21].

Several possible mechanisms have been suggested for the adverse effects of adiposity on CRC. First, adiposity tissue, especially visceral adipose can increase concentrations of serum insulin and insulin-like growth factor- I (IGF-I), and lead to leptin and adiponectin secretion, which play important roles in the pathogenesis of cancer [52]. In addition, inflammatory cytokine released by adiposity tissue and oxidative stress triggered by excess fat might involve in CRC tumorigenesis [47, 53]. However, the adjustment for inflammatory marker (i.e., WBC) in our study did not attenuate the association between obesity and incident CRC, suggesting that other pathogenic pathways, apart from WBC, might play a role. Future studies are warranted to confirm these results and elucidate the underlying mechanisms.

Previous studies comparing the predictive ability between BMI and abdominal obesity indices including WC and WHR in terms of their prediction for CRC risk showed that WC and WHR were more strongly associated with risk of CRC [54, 55]. However, there was no consensus on whether VAI/ABSI had better discrimination for predicting risk of CRC than conventional obesity indices. Our results showed that VAI was not superior to WC, WHR or WHtR in predicting CRC risk. A study pooling 11 Australian cohorts showed that ABSI had moderate discriminative ability for CRC with C-statistic values between 0.60 and 0.71 in both men and women after adjusting for age, education and smoking [29]. In a Swedish cohort study, ABSI showed a weaker association with colon cancer than WC in men and women [30]. However, ABSI but not WC predicted the risk of rectal cancer significantly in men [30]. None of the selected obesity indices was associated with CRC risk in women in this Swedish study [30]. Another study of postmenopausal women showed that the HR of CRC for ABSI appeared to be lower than those of WC, WHR and WHtR [22], although direct comparison of the HRs might be problematic because of different unites of the indices used for analysis. Results from previous studies as well as ours consistently showed that ABSI was not superior to other obesity indices in predicting CRC. Moreover, although a previous study showed that cancer risk prediction was improved by using both ABSI and BMI simultaneously [21], our results did not support the combination of ABSI and BMI in improving CRC prediction. As most of the new obesity indices such as VAI and ABSI were generated based on populations with different ethical background (i.e., VAI was derived from Caucasian [8] and ABSI from the US populations [9]), direct application of these indices to other populations might be limited. Note that different ethnic populations have different patterns of fat distribution, e.g., Asians were at greater risk of abdominal obesity and had low muscle mass than Caucasians [56, 57]. Whether the diverse fat distribution has differential effects on CRC risk remains to be explored in large scale prospective cohort studies with diverse ethnic populations.

The strengths of this study included a large sample size with long-term follow-up, the employment of standardized anthropometric measurements by well-trained nurses, which minimized the potential bias from self-reported value, and the comprehensive adjustment for a wide range of confounding factors. There were also some limitations that should be mentioned. First, as adiposity indices were assessed at a one-time point, they might have changed during follow-up. Further studies using repeated measurements of obesity indices might allow for more in-depth analysis. Second, we did not have the information of family CRC history [58], although we found no evidence that family CRC history plays a role in adiposity or fat distribution. Finally, our study was observational and could not confirm causal relation between obesity indices and CRC risk. However, interventional studies examining the effect of weight reduction on CRC risk require large sample size and long-term follow-up, and are unlikely to have good compliance, which limit the feasibility.

In conclusion, higher levels of BMI, WC, WHR, WHtR, and ABSI were associated with higher risk of CRC, and the associations were stronger for WC, WHR, WHtR and ABSI. Although clinical perspective focusing on abdominal obesity is appealing, VAI nor ABSI was unlikely an appropriate substitute for conventional adiposity indices in predicting CRC.

## Electronic supplementary material

Below is the link to the electronic supplementary material.

Supplementary Material 1

## Data Availability

Due to ethical restrictions protecting patient privacy, exposure data available on request from the Guangzhou Biobank Cohort Study Data Access Committee. Please contact us at gbcsdata@hku.hk for fielding data accession requests.
