# Supplementary material for: Association of novel and conventional obesity indices with colorectal cancer risk in older Chinese: a 14-year follow-up of the Guangzhou Biobank Cohort Study
Source: BMC Cancer. 2023 Mar 29;23:286. doi: 10.1186/s12885-023-10762-0 (PMC10053617; doi:10.1186/s12885-023-10762-0)
Supplement: Supplementary file 1 — Supplementary Material 1 [file 12885_2023_10762_MOESM1_ESM.docx]

**Supplementary information**

Supplementary Table 1. Pearson correlation between adiposity indices in the Guangzhou Biobank Cohort Study

Supplementary Table 2. Sensitivity analyses of association of adiposity indices with the risk of total colorectal cancer by excluding participants with less than 5 years of follow-up

Supplementary Table 3. Sensitivity analyses of association of adiposity indices with the risk of colon and rectal cancer by excluding participants with less than 5 years of follow-up

Supplementary Table 4. Sensitivity analyses of association of adiposity indices with the risk of total colorectal cancer on never-smokers

Supplementary Table 5. Sensitivity analyses of association of adiposity indices with the risk of colon and rectal cancer on never-smokers

Supplementary Table 6. Association of different combinations of body mass index and adiposity indices of central obesity with the risk of total colorectal cancer on 28 359 participants followed up from 2003–2008 (baseline) to April 2021 in the Guangzhou Biobank Cohort Study

Supplementary Table 7. Association of different combinations of body mass index and adiposity indices of central obesity with the risk of colon and rectal cancer on 28 359 participants followed up from 2003–2008 (baseline) to April 2021 in the Guangzhou Biobank Cohort Study

Supplementary Table 8. Harrell’ C statistic and 95% CI of combined assessment of obesity indices

Supplementary Table 9. Association of adiposity indices with the risk of colorectal cancer on 28 359 participants followed up from 2003–2008 (baseline) to April 2021 in the Guangzhou Biobank Cohort Study after additionally adjusting for white blood cell count

Supplementary Table 1. Pearson correlation between adiposity indices in the Guangzhou Biobank Cohort Study

| Man (n=7,848) |  |  |  |  |  |  |
| --- | --- | --- | --- | --- | --- | --- |
|  | BMI | WC | WHR | WHtR | VAI | ABSI |
| BMI | 1.00 | 0.82 | 0.95 | 0.84 | 0.26 | 0.56 |
| WC | | 1.00 | 0.83 | 0.60 | 0.24 | 0.66 |
| WHR | |  | 1.00 | 0.84 | 0.24 | 0.56 |
| WHtR | |  |  | 1.00 | 0.24 | 0.06 |
| VAI | | |  |  | 1.00 | 0.11 |
| ABSI | |  |  |  |  | 1.00 |
| Woman (n=20,511) |  |  |  |  |  |  |
|  | BMI | WC | WHR | WHtR | VAI | ABSI |
| BMI | 1.00 | 0.77 | 0.95 | 0.79 | 0.24 | 0.54 |
| WC | | 1.00 | 0.78 | 0.43 | 0.27 | 0.71 |
| WHR | |  | 1.00 | 0.78 | 0.23 | 0.55 |
| WHtR | |  |  | 1.00 | 0.17 | -0.06 |
| VAI | | |  |  | 1.00 | 0.16 |
| ABSI | |  |  |  |  | 1.00 |

Abbreviation: BMI: body mass index; WC: waist circumference; WHR: waist-to-hip ratio; WHtR: waist-to-height ratio; VAI: visceral adiposity index; ABSI: a body shape index

Supplementary Table 2. Sensitivity analyses of association of adiposity indices with the risk of colorectal cancer by excluding participants with less than 5 years of follow-up

|  | Incidence rate  (per 1000 person-years) | Crude  HR (95% CI) | Adjusted  HR (95% CI)^†^ |
| --- | --- | --- | --- |
| Body mass index (kg/m^2^) |  |  |  |
| <18.5 | 1.22 | 1.07 (0.68, 1.68) | 0.98 (0.62, 1.54) |
| ≥18.5 ~ <25.0 | 1.15 | 1.00 | 1.00 |
| ≥25.0 ~ <27.5 | 1.27 | 1.10 (0.88, 1.37) | 1.12 (0.89, 1.40) |
| ≥27.5 | 1.45 | 1.27 (0.98, 1.64) | 1.33 (1.02, 1.73)^*^ |
| z-score (1 SD=3.30) |  | 1.08 (0.99, 1.18) | 1.10 (1.01, 1.21)^*^ |
| Waist circumference (cm) |  |  |  |
| <80 for W;<90 for M | 1.14 | 1.00 | 1.00 |
| ≥80 for W; ≥90 for M | 1.40 | 1.19 (0.99, 1.43) | 1.26 (1.04, 1.53)^*^ |
| z-score (1 SD=8.69 for W;9.01 for M) |  | 1.21 (1.10, 1.32)^***^ | 1.17 (1.07, 1.28)^***^ |
| Waist-to-hip ratio |  |  |  |
| <0.8 for W;<0.9 for M | 1.03 | 1.00 | 1.00 |
| ≥0.8 for W; ≥0.9 for M | 1.28 | 1.25 (1.00, 1.56) | 1.45 (1.15, 1.84)^**^ |
| z-score (1 SD=0.07 for W;0.06 for W) |  | 1.25 (1.15, 1.36)^***^ | 1.20 (1.10, 1.31)^***^ |
| Waist-to-height ratio |  |  |  |
| <0.5 | 1.04 | 1.00 | 1.00 |
| ≥0.5 | 1.40 | 1.32 (1.10, 1.59)^**^ | 1.23 (1.02, 1.49)^*^ |
| z-score (1 SD=0.06) |  | 1.19 (1.09, 1.30)^***^ | 1.15 (1.06, 1.27)^**^ |
| Visceral adiposity index |  |  |  |
| Tertile 1 (<1.05) | 1.18 | 1.00 | 1.00 |
| Tertile 2 (≥1.05~<1.83) | 1.17 | 0.99 (0.79, 1.26) | 1.04 (0.83, 1.31) |
| Tertile 3 (≥1.83) | 1.31 | 1.12 (0.90,1.40) | 1.26 (1.00, 1.57)^*^ |
| z-score (1 SD=1.87) |  | 1.00 (0.91, 1.09) | 1.04 (0.95, 1.13) |
| A body shape index |  |  |  |
| Tertile 1 (<0.0741) | 0.87 | 1.00 | 1.00 |
| Tertile 2 (≥0.0741~<0.0783) | 1.25 | 1.43 (1.13, 1.82)^**^ | 1.19 (0.93, 1.52) |
| Tertile 3 (≥0.0783) | 1.55 | 1.80 (1.43, 2.26)^***^ | 1.29 (1.01, 1.65)^*^ |
| z-score (1 SD=0.0050) |  | 1.25 (1.16, 1.33)^***^ | 1.13 (1.03, 1.24)^**^ |

†Adjusting for age, sex, smoking, alcohol drinking, household annual income, education, physical activity, intake of vegetable, fruits and red meat

*: P<0.05; **: P<0.01; ***: P<0.001

Supplementary Table 3. Sensitivity analyses of association of adiposity indices with the risk of colon and rectal cancer by excluding participants with less than 5 years of follow-up

| Cancer type | Colon cancer | | | Rectal cancer | | |
| --- | --- | --- | --- | --- | --- | --- |
|  | Incidence rate  (per 1000 person-years) | Crude  HR (95% CI) | Adjusted  HR (95% CI)^†^ | Incidence rate  (per 1000 person-years) | Crude  HR (95% CI) | Adjusted  HR (95% CI)^†^ |
| Body mass index (kg/m^2^) |  |  |  |  |  |  |
| <18.5 | 0.98 | 1.27 (0.76, 2.11) | 1.14 (0.69, 1.91) | 0.24 | 0.66 (0.24, 1.79) | 0.62 (0.23, 1.69) |
| ≥18.5 ~ <25.0 | 0.78 | 1.00 | 1.00 | 0.37 | 1.00 | 1.00 |
| ≥25.0 ~ <27.5 | 0.82 | 1.05 (0.80, 1.39) | 1.07 (0.81, 1.42) | 0.45 | 1.19 (0.81, 1.75) | 1.21 (0.82, 1.78) |
| ≥27.5 | 1.03 | 1.33 (0.98, 1.81) | 1.41 (1.03, 1.92)^*^ | 0.42 | 1.14 (0.71, 1.83) | 1.18 (0.73, 1.91) |
| z-score (1 SD=3.30) |  | 1.10 (0.98, 1.22) | 1.13 (1.01, 1.25)^*^ |  | 1.03 (0.88, 1.22) | 1.06 (0.90, 1.24) |
| Waist circumference (cm) |  |  |  |  |  |  |
| <80 for W;<90 for M | 0.75 | 1.00 | 1.00 | 0.39 | 1.00 | 1.00 |
| ≥80 for W; ≥90 for M | 0.97 | 1.29 (1.04, 1.62)^*^ | 1.35 (1.07, 1.71)^*^ | 0.39 | 0.98 (0.70, 1.38) | 1.08 (0.76, 1.53) |
| z score (1 SD=8.69 for W;9.01 for M) |  | 1.25 (1.12, 1.39)^***^ | 1.20 (1.08, 1.34)^**^ |  | 1.12 (0.96, 1.31) | 1.10 (0.94, 1.29) |
| Waist-to-hip ratio |  |  |  |  |  |  |
| <0.8 for W;<0.9 for M | 0.73 | 1.00 | 1.00 | 0.40 | 1.00 | 1.00 |
| ≥0.8 for W; ≥0.9 for M | 0.86 | 1.33 (1.01, 1.74)^*^ | 1.52 (1.14, 2.03)^**^ | 0.39 | 1.10 (0.75, 1.61) | 1.33 (0.90, 1.99) |
| z score (1 SD=0.07 for W;0.06 for M) |  | 1.30 (1.18, 1.43)^***^ | 1.24 (1.12, 1.38)^***^ |  | 1.14 (0.98, 1.33) | 1.11 (0.95, 1.30) |
| Waist-to-height ratio |  |  |  |  |  |  |
| <0.5 | 0.69 | 1.00 | 1.00 | 0.34 | 1.00 | 1.00 |
| ≥0.5 | 0.94 | 1.35 (1.08, 1.69)^**^ | 1.22 (0.97, 1.53) | 0.43 | 1.27 (0.91, 1.75) | 1.25 (0.90, 1.75) |
| z-score (1 SD=0.06) |  | 1.25 (1.12, 1.39)^***^ | 1.19 (1.07, 1.33)^**^ |  | 1.08 (0.92, 1.27) | 1.07 (0.91, 1.27) |
| Visceral adiposity index |  |  |  |  |  |  |
| Tertile 1 (<1.05) | 0.76 | 1.00 | 1.00 | 0.42 | 1.00 | 1.00 |
| Tertile 2 (≥1.05~<1.83) | 0.80 | 1.05 (0.79, 1.38) | 1.10 (0.83, 1.45) | 0.37 | 0.88 (0.60, 1.30) | 0.94 (0.64, 1.40) |
| Tertile 3 (≥1.83) | 0.93 | 1.23 (0.94, 1.61) | 1.38 (1.05, 1.82)^*^ | 0.39 | 0.93 (0.63, 1.36) | 1.04 (0.70, 1.53) |
| z-score (1 SD=1.87) |  | 1.04 (0.95, 1.14) | 1.08 (0.99, 1.18) |  | 0.87 (0.70, 1.08) | 0.91 (0.74, 1.12) |
| A body shape index |  |  |  |  |  |  |
| Tertile 1 (<0.0742) | 0.57 | 1.00 | 1.00 | 0.30 | 1.00 | 1.00 |
| Tertile 2 (≥0.0742~<0.0784) | 0.83 | 1.45 (1.08, 1.94)^*^ | 1.16 (0.86, 1.57) | 0.42 | 1.41 (0.93, 2.12) | 1.24 (0.81, 1.89) |
| Tertile 3 (≥0.0784) | 1.09 | 1.92 (1.45, 2.55)^***^ | 1.32 (0.97, 1.78) | 0.46 | 1.56 (1.04, 2.34)^*^ | 1.23 (0.80, 1.91) |
| z-score (1 SD=0.0050) |  | 1.28 (1.18, 1.38)^***^ | 1.16 (1.04, 1.30)^**^ |  | 1.16 (1.01, 1.34)^*^ | 1.07 (0.90, 1.26) |

†Adjusting for age, sex, smoking, alcohol drinking, household annual income, education, physical activity, intake of vegetable, fruits and red meat

*: P<0.05; **: P<0.01; ***: P<0.001

Supplementary Table 4. Sensitivity analyses of association of adiposity indices with the risk of colorectal cancer on never-smokers

|  | Incidence rate  (per 1000 person-years) | Crude  HR (95% CI) | Adjusted  HR (95% CI)^†^ |
| --- | --- | --- | --- |
| Body mass index (kg/m^2^) |  |  |  |
| <18.5 | 1.56 | 1.14 (0.72, 1.80) | 1.06 (0.67, 1.67) |
| ≥18.5 ~ <25.0 | 1.37 | 1.00 | 1.00 |
| ≥25.0 ~ <27.5 | 1.54 | 1.12 (0.90, 1.40) | 1.12 (0.90, 1.40) |
| ≥27.5 | 1.53 | 1.12 (0.86, 1.47) | 1.14 (0.87, 1.49) |
| z-score (1 SD=3.31) |  | 1.07 (0.98, 1.17) | 1.08 (0.99, 1.19) |
| Waist circumference (cm) |  |  |  |
| <80 for W;<90 for M | 1.34 | 1.00 | 1.00 |
| ≥80 for W; ≥90 for M | 1.60 | 1.20 (0.99, 1.44) | 1.18 (0.98, 1.43) |
| z-score (1 SD=8.68 for W;8.72 for M) |  | 1.24 (1.13, 1.35)^***^ | 1.18 (1.08, 1.30)^***^ |
| Waist-to-hip ratio |  |  |  |
| <0.8 for W;<0.9 for M | 1.12 | 1.00 | 1.00 |
| ≥0.8 for W; ≥0.9 for M | 1.52 | 1.36 (1.07, 1.74) ^*^ | 1.48 (1.15, 1.92)^**^ |
| z-score (1 SD=0.07 for W;0.06 for W) |  | 1.24 (1.14, 1.34)^***^ | 1.17 (1.07, 1.28)^***^ |
| Waist-to-height ratio |  |  |  |
| <0.5 | 1.24 | 1.00 | 1.00 |
| ≥0.5 | 1.60 | 1.29 (1.07, 1.55)^**^ | 1.14 (0.95, 1.39) |
| z-score (1 SD=0.06) |  | 1.21 (1.11, 1.32)^***^ | 1.14 (1.04, 1.26)^**^ |
| Visceral adiposity index |  |  |  |
| Tertile 1 (<1.09) | 1.30 | 1.00 | 1.00 |
| Tertile 2 (≥1.09~<1.89) | 1.35 | 1.04 (0.82, 1.31) | 1.04 (0.83, 1.33) |
| Tertile 3 (≥1.89) | 1.64 | 1.27 (1.01,1.59)^*^ | 1.34 (1.07, 1.69)^*^ |
| z-score (1 SD=1.88) |  | 1.03 (0.95, 1.12) | 1.06 (0.98, 1.14) |
| A body shape index |  |  |  |
| Tertile 1 (<0.0738) | 1.03 | 1.00 | 1.00 |
| Tertile 2 (≥0.0738~<0.0779) | 1.48 | 1.52 (1.19, 1.93)^***^ | 1.30 (1.02, 1.66)^*^ |
| Tertile 3 (≥0.0779) | 1.95 | 2.01 (1.60, 2.53)^***^ | 1.49 (1.16, 1.91)^**^ |
| z-score (1 SD=0.0050) |  | 1.26 (1.18, 1.34)^***^ | 1.16 (1.06, 1.27)^***^ |

†Adjusting for age, sex, alcohol drinking, household annual income, education, physical activity, intake of vegetable, fruits and red meat

*: P<0.05; **: P<0.01; ***: P<0.001

Supplementary Table 5. Sensitivity analyses of association of adiposity indices with the risk of colon and rectal cancer on never-smokers

| Cancer type | Colon cancer | | | Rectal cancer | | |
| --- | --- | --- | --- | --- | --- | --- |
|  | Incidence rate  (per 1000 person-years) | Crude  HR (95% CI) | Adjusted  HR (95% CI)^†^ | Incidence rate  (per 1000 person-years) | Crude  HR (95% CI) | Adjusted  HR (95% CI)^†^ |
| Body mass index (kg/m^2^) |  |  |  |  |  |  |
| <18.5 | 1.14 | 1.07 (0.67, 1.70) | 0.96 (0.60, 1.53) | 0.62 | 1.53 (0.74, 3.16) | 1.46 (0.71, 3.03) |
| ≥18.5 ~ <25.0 | 1.07 | 1.00 | 1.00 | 0.41 | 1.00 | 1.00 |
| ≥25.0 ~ <27.5 | 1.00 | 0.94 (0.73, 1.20) | 0.95 (0.74, 1.21) | 0.55 | 1.34 (0.91, 1.96) | 1.32 (0.90, 1.95) |
| ≥27.5 | 1.26 | 1.18 (0.89, 1.55) | 1.23 (0.93, 1.62) | 0.39 | 0.97 (0.57, 1.63) | 0.97 (0.57, 1.64) |
| z-score (1 SD=3.31) |  | 1.08 (0.98, 1.18) | 1.11 (1.01, 1.21)^*^ |  | 1.01 (0.86, 1.19) | 1.01 (0.86, 1.20) |
| Waist circumference (cm) |  |  |  |  |  |  |
| <80 for W;<90 for M | 1.02 | 1.00 | 1.00 | 0.44 | 1.00 | 1.00 |
| ≥80 for W; ≥90 for M | 1.21 | 1.18 (0.97, 1.44) | 1.23 (1.00, 1.50) | 0.45 | 1.02 (0.73, 1.43) | 1.00 (0.71, 1.42) |
| z score (1 SD=8.86 for W;8.72 for M) |  | 1.23 (1.13, 1.36)^***^ | 1.18 (1.07, 1.29)^***^ |  | 1.15 (0.98, 1.35) | 1.11 (0.94, 1.31) |
| Waist-to-hip ratio |  |  |  |  |  |  |
| <0.8 for W;<0.9 for M | 0.87 | 1.00 | 1.00 | 0.41 | 1.00 | 1.00 |
| ≥0.8 for W; ≥0.9 for M | 1.15 | 1.32 (1.04, 1.68)^*^ | 1.53 (1.19, 1.97)^***^ | 0.46 | 1.12 (0.74, 1.69) | 1.18 (0.77, 1.83) |
| z score (1 SD=0.07 for W;0.06 for M) |  | 1.28 (1.18, 1.39)^***^ | 1.21 (1.10, 1.33)^***^ |  | 1.11 (0.94, 1.30) | 1.06 (0.90, 1.25) |
| Waist-to-height ratio |  |  |  |  |  |  |
| <0.5 | 0.93 | 1.00 | 1.00 | 0.40 | 1.00 | 1.00 |
| ≥0.5 | 1.21 | 1.30 (1.07, 1.58)^**^ | 1.16 (0.95, 1.42) | 0.48 | 1.20 (0.86, 1.67) | 1.10 (0.78, 1.55) |
| z-score (1 SD=0.06) |  | 1.23 (1.12, 1.35)^***^ | 1.16 (1.06, 1.28)^**^ |  | 1.10 (0.93, 1.29) | 1.05 (0.89, 1.25) |
| Visceral adiposity index |  |  |  |  |  |  |
| Tertile 1 (<1.05) | 1.02 | 1.00 | 1.00 | 0.41 | 1.00 | 1.00 |
| Tertile 2 (≥1.05~<1.83) | 1.06 | 1.03 (0.81, 1.31) | 1.10 (0.86, 1.40) | 0.40 | 0.97 (0.63, 1.47) | 0.97 (0.63, 1.49) |
| Tertile 3 (≥1.83) | 1.18 | 1.16 (0.92, 1.46) | 1.32 (1.04, 1.67)^*^ | 0.52 | 1.24 (0.84, 1.85) | 1.29 (0.86, 1.93) |
| z-score (1 SD=1.88) |  | 1.01 (0.93, 1.11) | 1.06 (0.97, 1.15) |  | 1.00 (0.85, 1.17) | 1.01 (0.86, 1.19) |
| A body shape index |  |  |  |  |  |  |
| Tertile 1 (<0.0742) | 0.74 | 1.00 | 1.00 | 0.32 | 1.00 | 1.00 |
| Tertile 2 (≥0.0742~<0.0784) | 1.03 | 1.40 (1.08, 1.82)^*^ | 1.11 (0.85, 1.45) | 0.47 | 1.47 (0.97, 2.25) | 1.35 (0.88, 2.07) |
| Tertile 3 (≥0.0784) | 1.50 | 2.05 (1.60, 2.61)^***^ | 1.36 (1.05, 1.76)^*^ | 0.57 | 1.78 (1.18, 2.70)^**^ | 1.49 (0.95, 2.32) |
| z-score (1 SD=0.0050) |  | 1.28 (1.20, 1.36)^***^ | 1.15 (1.04, 1.26)^**^ |  | 1.20 (1.05, 1.38)^**^ | 1.13 (0.96, 1.33) |

†Adjusting for age, sex, alcohol drinking, household annual income, education, physical activity, intake of vegetable, fruits and red meat

*: P<0.05; **: P<0.01; ***: P<0.001

Supplementary Table 6. Association of different combinations of body mass index and adiposity indices of centraobesity with the risk of total colorectal cancer on 28 359 participants followed up from 2003–2008 (baseline) to April 2021 in the Guangzhou Biobank Cohort Study

|  | Incidence rate  (per 1000 person-years) | Crude  HR (95% CI) | Adjusted  HR (95% CI)^†^ |
| --- | --- | --- | --- |
| BMI (kg/m^2^) & WC (cm)^‡^ |  |  |  |
| BMI<27.5 & WC<80(W)/ <90(M) | 1.54 | 1.00 | 1.00 |
| BMI≥27.5 & WC<80(W)/ <90(M) | 1.15 | 0.75 (0.31, 1.81) | 0.76 (0.32, 1.84) |
| BMI<27.5 & WC≥80(W)/ ≥90(M) | 1.67 | 1.08 (0.90, 1.31) | 1.13 (0.93, 1.37) |
| BMI≥27.5 & WC≥80(W)/ ≥90(M) | 1.84 | 1.19 (0.94, 1.51) | 1.27 (1.00, 1.62)^*^ |
| BMI (kg/m^2^) & WHR^‡^ |  |  |  |
| BMI<27.5 & WHR<0.8(W)/ <0.9(M) | 1.40 | 1.00 | 1.00 |
| BMI≥27.5 & WHR<0.8(W)/ <0.9(M) | 1.11 | 0.79 (0.25, 2.48) | 0.82 (0.26, 2.59) |
| BMI<27.5 & WHR≥0.8(W)/ ≥0.9(M) | 1.65 | 1.17 (0.96, 1.43) | 1.37 (1.11, 1.68)^**^ |
| BMI≥27.5 & WHR≥0.8(W)/ ≥0.9(M) | 1.82 | 1.30 (0.99, 1.70) | 1.54 (1.16, 2.04)^**^ |
| BMI (kg/m^2^) & WHtR^‡^ |  |  |  |
| BMI<27.5 & WHtR<0.5 | 1.41 | 1.00 | 1.00 |
| BMI≥27.5 & WHtR<0.5 | 0.00 | - | - |
| BMI<27.5 & WHtR≥0.5 | 1.76 | 1.25 (1.06, 1.48)^**^ | 1.12 (0.94, 1.33) |
| BMI≥27.5 & WHtR≥0.5 | 1.81 | 1.29 (1.02, 1.65)^*^ | 1.28 (1.00, 1.63) |
| BMI (kg/m^2^) & VAI^‡^ |  |  |  |
| BMI<27.5 & VAI<1.83 | 1.52 | 1.00 | 1.00 |
| BMI≥27.5 & VAI<1.83 | 1.74 | 1.14 (0.84, 1.57) | 1.18 (0.86, 1.62) |
| BMI<27.5 & VAI≥1.83 | 1.71 | 1.13 (0.94, 1.35) | 1.23 (1.03, 1.48)^*^ |
| BMI≥27.5 & VAI≥1.83 | 1.82 | 1.21 (0.88, 1.64) | 1.36 (1.00, 1.86) |
| BMI (kg/m^2^) & ABSI^‡^ |  |  |  |
| BMI<27.5 & ABSI<0.0784 | 1.30 | 1.00 | 1.00 |
| BMI≥27.5 & ABSI<0.0784 | 1.54 | 1.19 (0.89, 1.59) | 1.23 (0.92, 1.66) |
| BMI<27.5 & ABSI≥0.0784 | 2.14 | 1.66 (1.40, 1.96)^***^ | 1.26 (1.06, 1.51)^**^ |
| BMI≥27.5 & ABSI≥0.0784 | 2.29 | 1.77 (1.25, 2.50)^**^ | 1.41 (0.99, 2.00) |

^‡^Abbreviations: BMI (body mass index), WC (waist circumference), WHR (waist-to-hip ratio), WHtR (waist-to-height ratio), VAI (visceral adiposity index), ABSI (a body shape index)

^†^Adjusting for age, sex, smoking, alcohol drinking, household annual income, education, physical activity, intake of vegetable, fruits and red meat.

^*^: *P*<0.05; ^**^: *P*<0.01; ^***^: *P*<0.001.

Supplementary Table 7. Association of different combinations of body mass index and adiposity indices of central obesity with the risk of colon and rectal cancer on 28 359 participants followed up from 2003–2008 (baseline) to April 2021 in the Guangzhou Biobank Cohort Study

| Cancer type | Colon cancer | | | Rectal cancer | | |
| --- | --- | --- | --- | --- | --- | --- |
|  | Incidence rate  (per 1000 person-years) | Crude  HR (95% CI) | Adjusted  HR (95% CI)^†^ | Incidence rate  (per 1000 person-years) | Crude  HR (95% CI) | Adjusted  HR (95% CI)^†^ |
| BMI (kg/m^2^) & WC (cm)^‡^ |  |  |  |  |  |  |
| BMI<27.5 & WC<80(W)/ <90(M) | 1.02 | 1.00 | 1.00 | 0.52 | 1.00 | 1.00 |
| BMI≥27.5 & WC<80(W)/ <90(M) | 0.92 | 0.91 (0.34, 2.44) | 0.93 (0.34, 2.49) | 0.23 | 0.44 (0.06, 3.16) | 0.45 (0.06, 3.21) |
| BMI<27.5 & WC≥80(W)/ ≥90(M) | 1.16 | 1.14 (0.91, 1.43) | 1.16 (0.92, 1.47) | 0.51 | 0.98 (0.70, 1.37) | 1.06 (0.75, 1.51) |
| BMI≥27.5 & WC≥80(W)/ ≥90(M) | 1.29 | 1.27 (0.95, 1.68) | 1.34 (1.01, 1.79)^*^ | 0.55 | 1.05 (0.68, 1.61) | 1.13 (0.73, 1.75) |
| BMI (kg/m^2^) & WHR^‡^ |  |  |  |  |  |  |
| BMI<27.5 & WHR<0.8(W)/ <0.9(M) | 0.87 | 1.00 | 1.00 | 0.53 | 1.00 | 1.00 |
| BMI≥27.5 & WHR<0.8(W)/ <0.9(M) | 0.74 | 0.84 (0.21, 3.43) | 0.88 (0.22, 3.57) | 0.37 | 0.70 (0.10, 5.07) | 0.74 (0.10, 5.37) |
| BMI<27.5 & WHR≥0.8(W)/ ≥0.9(M) | 1.13 | 1.29 (1.01, 1.64)^*^ | 1.49 (1.15, 1.92)^**^ | 0.52 | 0.98 (0.71, 1.36) | 1.17 (0.82, 1.65) |
| BMI≥27.5 & WHR≥0.8(W)/ ≥0.9(M) | 1.29 | 1.47 (1.06, 2.05)^*^ | 1.74 (1.24, 2.45)^**^ | 0.53 | 1.00 (0.62, 1.62) | 1.20 (0.73, 1.98) |
| BMI (kg/m^2^) & WHtR^‡^ |  |  |  |  |  |  |
| BMI<27.5 & WHtR<0.5 | 0.93 | 1.00 | 1.00 | 0.47 | 1.00 | 1.00 |
| BMI≥27.5 & WHtR<0.5 | 0.00 | - | - | 0.00 | - | - |
| BMI<27.5 & WHtR≥0.5 | 1.19 | 1.27 (1.03, 1.56)^*^ | 1.11 (0.90, 1.37) | 0.57 | 1.22 (0.91, 1.63) | 1.16 (0.86, 1.56) |
| BMI≥27.5 & WHtR≥0.5 | 1.28 | 1.38 (1.03, 1.84)^*^ | 1.34 (1.00, 1.79) | 0.53 | 1.13 (0.73, 1.76) | 1.15 (0.74, 1.80) |
| BMI (kg/m^2^) & VAI^‡^ |  |  |  |  |  |  |
| BMI<27.5 & VAI<1.83 | 1.02 | 1.00 | 1.00 | 0.50 | 1.00 | 1.00 |
| BMI≥27.5 & VAI<1.83 | 1.22 | 1.20 (0.83, 1.75) | 1.24 (0.85, 1.80) | 0.51 | 1.02 (0.58, 1.81) | 1.07 (0.60, 1.90) |
| BMI<27.5 & VAI≥1.83 | 1.15 | 1.14 (0.91, 1.41) | 1.24 (1.00, 1.55) | 0.56 | 1.11 (0.81, 1.51) | 1.21 (0.88, 1.66) |
| BMI≥27.5 & VAI≥1.83 | 1.29 | 1.28 (0.89, 1.85) | 1.46 (1.01, 2.12)^*^ | 0.53 | 1.05 (0.59, 1.86) | 1.18 (0.66, 2.10) |
| BMI (kg/m^2^) & ABSI^‡^ |  |  |  |  |  |  |
| BMI<27.5 & ABSI<0.0784 | 0.86 | 1.00 | 1.00 | 0.44 | 1.00 | 1.00 |
| BMI≥27.5 & ABSI<0.0784 | 1.05 | 1.23 (0.86, 1.75) | 1.26 (0.89, 1.81) | 0.50 | 1.12 (0.67, 1.88) | 1.18 (0.70, 1.97) |
| BMI<27.5 & ABSI≥0.0784 | 1.47 | 1.72 (1.39, 2.11)^***^ | 1.28 (1.03, 1.58)^*^ | 0.68 | 1.54 (1.14, 2.07)^**^ | 1.24 (0.91, 1.70) |
| BMI≥27.5 & ABSI≥0.0784 | 1.72 | 2.01 (1.35, 3.01)^***^ | 1.57 (1.04, 2.36)^*^ | 0.57 | 1.30 (0.66, 2.58) | 1.09 (0.54, 2.17) |

^‡^Abbreviations: BMI (body mass index), WC (waist circumference), WHR (waist-to-hip ratio), WHtR (waist-to-height ratio), VAI (visceral adiposity index), ABSI (a body shape index)

^†^Adjusting for age, sex, smoking, alcohol drinking, household annual income, education, physical activity, intake of vegetable, fruits and red meat.

^*^: *P*<0.05; ^**^: *P*<0.01; ^***^: *P*<0.001.

Supplementary Table 8. Harrell’ C statistic and 95% CI of combined assessment of obesity indices

|  | Harrell’s C statistic (95% CI) for combination of obesity indices | | |
| --- | --- | --- | --- |
|  | Colorectal cancer | Colon cancer | Rectal cancer |
| Body mass index & Waist circumference | 0.642 (0.620, 0.664) | 0.660 (0.635, 0.685) | 0.621 (0.582, 0.660) |
| Body mass index & Waist-to-hip ratio | 0.645 (0.623, 0.667) | 0.665 (0.640, 0.690) | 0.621 (0.582, 0.660) |
| Body mass index & Waist-to-height ratio | 0.641 (0.619, 0.663) | 0.660 (0.635, 0.685) | 0.621 (0.580, 0.662) |
| Body mass index & Visceral adiposity index | 0.644 (0.622, 0.666) | 0.663 (0.638, 0.688) | 0.623 (0.582, 0.664) |
| Body mass index & A body shape index | 0.644 (0.622, 0.666) | 0.662 (0.637, 0.687) | 0.623 (0.582, 0.664) |

All models were adjusted for age, sex, smoking, alcohol drinking, household annual income, education, physical activity, intake of vegetable, fruits and red meat.

Supplementary Table 9. Association of adiposity indices with the risk of colorectal cancer on 28 359 participants followed up from 2003–2008 (baseline) to April 2021 in the Guangzhou Biobank Cohort Study after additionally adjusting for white blood cell count.

| Cancer type | Colorectal cancer | Colon cancer | Rectal cancer |
| --- | --- | --- | --- |
|  | Adjusted  HR (95% CI)^†^ | Adjusted  HR (95% CI)^†^ | Adjusted  HR (95% CI)^†^ |
| Body mass index (kg/m^2^) |  |  |  |
| <18.5 | 0.98 (0.67, 1.45) | 0.97 (0.61, 1.55) | 1.01 (0.51, 2.00) |
| ≥18.5 ~ <25.0 | 1.00 | 1.00 | 1.00 |
| ≥25.0 ~ <27.5 | 1.05 (0.86, 1.28) | 0.94 (0.73, 1.21) | 1.29 (0.92, 1.79) |
| ≥27.5 | 1.19 (0.94, 1.50) | 1.22 (0.92, 1.61) | 1.12 (0.73, 1.73) |
| z-score (1 SD=3.31) | 1.08 (1.00, 1.17) | 1.10 (1.00, 1.21)^*^ | 1.03 (0.90, 1.19) |
| Waist circumference (cm) |  |  |  |
| <80 for W;<90 for M | 1.00 | 1.00 | 1.00 |
| ≥80 for W; ≥90 for M | 1.17 (0.99, 1.39) | 1.22 (0.99, 1.50) | 1.09 (0.80, 1.48) |
| z score (1 SD=8.70 for W;9.08 for M) | 1.15 (1.06, 1.24)^***^ | 1.18 (1.07, 1.30)^**^ | 1.09 (0.95, 1.26) |
| Waist-to-hip ratio |  |  |  |
| <0.8 for W;<0.9 for M | 1.00 | 1.00 | 1.00 |
| ≥0.8 for W; ≥0.9 for M | 1.39 (1.14, 1.71)^**^ | 1.53 (1.19, 1.97)^**^ | 1.18 (0.84, 1.65) |
| z score (1 SD=0.07 for W;0.06 for M) | 1.16 (1.08, 1.26)^***^ | 1.21 (1.10, 1.33)^***^ | 1.07 (0.92, 1.23) |
| Waist-to-height ratio |  |  |  |
| <0.5 | 1.00 | 1.00 | 1.00 |
| ≥0.5 | 1.15 (0.98, 1.36) | 1.15 (0.94, 1.41) | 1.16 (0.86, 1.55) |
| z-score (1 SD=0.06) | 1.13 (1.04, 1.23)^**^ | 1.16 (1.05, 1.29)^**^ | 1.06 (0.92, 1.23) |
| Visceral adiposity index |  |  |  |
| Tertile 1 (<1.05) | 1.00 | 1.00 | 1.00 |
| Tertile 2 (≥1.05~<1.83) | 1.02 (0.84, 1.24) | 1.09 (0.86, 1.39) | 0.88 (0.62, 1.25) |
| Tertile 3 (≥1.83) | 1.24 (1.02, 1.52)^*^ | 1.31 (1.02, 1.67)^*^ | 1.12 (0.80, 1.58) |
| z-score (1 SD=1.85) | 1.03 (0.96, 1.12) | 1.05 (0.97, 1.15) | 0.98 (0.84, 1.15) |
| A body shape index |  |  |  |
| Tertile 1 (<0.0742) | 1.00 | 1.00 | 1.00 |
| Tertile 2 (≥0.0742~<0.0784) | 1.13 (0.91, 1.40) | 1.11 (0.85, 1.45) | 1.17 (0.80, 1.70) |
| Tertile 3 (≥0.0784) | 1.34 (1.08, 1.66)^**^ | 1.35 (1.04, 1.76)^*^ | 1.32 (0.90, 1.92) |
| z-score (1 SD=0.0050) | 1.12 (1.04, 1.22)^**^ | 1.14 (1.04, 1.26)^**^ | 1.08 (0.94, 1.25) |

^†^Adjusting for age, sex, smoking, alcohol drinking, household annual income, education, physical activity, intake of vegetable, fruits, red meat and white blood cell count.

^*^: *P*<0.05; ^**^: *P*<0.01; ^***^: *P*<0.001.
